# Identification of candidate genes for human pituitary development by EST analysis

**DOI:** 10.1186/1471-2164-10-109

**Published:** 2009-03-15

**Authors:** Yueyun Ma, Xiaofei Qi, Jianjun Du, Shaojun Song, Dongyun Feng, Jia Qi, Zhidong Zhu, Xin Zhang, Huasheng Xiao, Zeguang Han, Xiaoke Hao

**Affiliations:** 1 Center for Clinical Laboratory Medicine of PLA, Xijing Hospital, Fourth Military Medical University, Xi'an 710032, PR China; 2 Chinese National Human Genome Center at Shanghai, 351 Guo Shou-Jing Road, Shanghai 201203, PR China; 3 Department of General Surgery, Xijing Hospital, Fourth Military Medical University, Xi'an 710032, PR China; 4 Department of Neurology, Xijing Hospital, Fourth Military Medical University, Xi'an 710032, PR China

## Abstract

**Background:**

The pituitary is a critical neuroendocrine gland that is comprised of five hormone-secreting cell types, which develops in tandem during the embryonic stage. Some essential genes have been identified in the early stage of adenohypophysial development, such as PITX1, FGF8, BMP4 and SF-1. However, it is likely that a large number of signaling molecules and transcription factors essential for determination and terminal differentiation of specific cell types remain unidentified. High-throughput methods such as microarray analysis may facilitate the measurement of gene transcriptional levels, while Expressed sequence tag (EST) sequencing, an efficient method for gene discovery and expression level analysis, may no-redundantly help to understand gene expression patterns during development.

**Results:**

A total of 9,271 ESTs were generated from both fetal and adult pituitaries, and assigned into 961 gene/EST clusters in fetal and 2,747 in adult pituitary by homology analysis. The transcription maps derived from these data indicated that developmentally relevant genes, such as Sox4, ST13 and ZNF185, were dominant in the cDNA library of fetal pituitary, while hormones and hormone-associated genes, such as GH1, GH2, POMC, LHβ, CHGA and CHGB, were dominant in adult pituitary. Furthermore, by using RT-PCR and *in situ *hybridization, Sox4 was found to be one of the main transcription factors expressed in fetal pituitary for the first time. It was expressed at least at E12.5, but decreased after E17.5. In addition, 40 novel ESTs were identified specifically in this tissue.

**Conclusion:**

The significant changes in gene expression in both tissues suggest a distinct and dynamic switch between embryonic and adult pituitaries. All these data along with Sox4 should be confirmed to further understand the community of multiple signaling pathways that act as a cooperative network that regulates maturation of the pituitary. It was also suggested that EST sequencing is an efficient means of gene discovery.

## Background

The pituitary is well known as one of the most important glands in the endocrine system, and secretes hormones that orchestrate many physiological processes such as growth, sexual development, metabolism and the stress response. It contains three lobes and is of dual embryonic origin [1]. The posterior lobe is of neuroectodermal origin while the anterior and intermediate lobes are of ectodermal origin. The anterior pituitary gland consists of five cell types: somatotrophs [producing growth hormone (GH)], lactotrophs [producing prolactin (PRL)], gonadotrophs [producing luteinizing hormone (LH) and follicle stimulating hormone (FSH)], thyrotrophs [producing thyroid stimulating hormone (TSH)], and corticotrophs [producing adrenocorticotropic hormone (ACTH), proteolytically cleaved from proopiomelanocortin (POMC)]. Melanotrophs [producing melanocyte-stimulating hormone (MSH)], proteolytically cleaved from POMC are the sixth hormone-producing cell type, but these are situated within the intermediate lobe. During embryonic development, the six specific cell types arise from Rathke's pouch (RP), and form the anterior and intermediate lobes under the control of various mechanisms [2].

In the past several years, most aspects of pituitary development have been extensively explored, because of the relatively simple structure of the gland and its critical role in the neuroendocrine system [3]. According to the structural changes and cell-type-specific occurrence, development and differentiation of the pituitary can be divided into three major stages [4,5]. The onset of pituitary organogenesis is marked by a striking restriction of Shh, BMP4, FGF8 and Wnt5a, which are uniformly expressed in the oral ectoderm from the invaginating RP. In murine embryos, this is formed around E9.0, which results in a molecular boundary in the oral ectoderm [6,7]. The second phase of pituitary development appears to involve BMP2, Wnt4, and Prop1 expression, which participate in the progression from organ commitment to cell-type determination that occurs between E10 and E12. At this time, the RP extends dorsally and expands to form a flattened epithelium close to the infundibular recess of the diencephalon. Hes1 and Hes5 have been identified to play a role in controlling the progenitor pool, intermediate lobe specification, and posterior lobe formation during pituitary development [8]. Terminal differentiation of the hormone-secreting cells occurs after E12 and is marked by the expression of LH and FSH in gonadotrophs, GH in somatotrophs, and PRL in lactotrophs. Transcription factors like Prop1, TBX19, Gata2 and POU1F1 are necessary for the terminal differentiation events and cell-type-specific gene expression [9].

Three recent studies using cDNA microarray have been carried out to profile gene expression pattern during pituitary development [10-12]. Many genes, such as carboxypeptidase E (CPE), stathmin (STMN1), endothelin type A receptor (EDNRA), dual-specificity phosphatase 1 (DUSP1), FGF2, FGF4, ZHX1B, DUSP3 and Sox10, were newly identified as being involved in the determination and cell-type-specific gene expression of the six mature endocrine cell types. It has been suggested that a large number of signaling molecules and transcription factors essential for pituitary development remain unclear, especially at the end of differentiation. All these large-scale studies were carried out based on neuroendocrine-system-specific cDNA microarrays. In addition, it is necessary to point out that the understanding of pituitary development and the regulatory programs that control hormone expression have been mostly derived from animal experiments, in particular mice and chickens. Accumulating evidence has implied that there are vital differences in gene expression profiles between development in mice and humans [13,14]. Until now, there has been a lack of information from expressed sequence tag (EST) libraries of human fetal pituitary.

For a comprehensive insight into the molecular mechanisms involved in human pituitary development, we used a relatively large number of ESTs to investigate the differentially expressed genes/ESTs in fetal and adult pituitaries. In addition to data from microarrays, the EST data reported herein will lead to an additional understanding of human pituitary development and hormone expression.

## Results

### General characteristics of ESTs derived from human pituitaries

**Figure 1:**
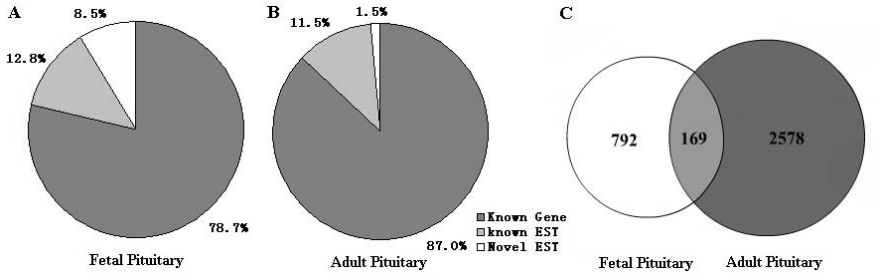
**Homologous analysis of EST clusters from human pituitaries**. (A) 961 EST clusters were generated from fetal pituitary using software CAT 3.5 (Pangea) with default parameters, and classified as the known genes, known ESTs and novel ESTs by searching against public DNA and protein databases. Novel EST clusters indicated that no known human genes and ESTs in public databases shared identity with the ESTs. (B) Distribution of 2747 EST clusters from adult pituitary based on homology analysis. (C) Overlapping of EST clusters from fetal and adult pituitaries. Overlapping region indicates the numbers of EST clusters shared by both cDNA resources, while the other numbers represent EST clusters found only in fetal or adult cDNA libraries.

To profile the gene expression of fetal pituitary, 4,172 clones from the cDNA library were randomly selected and sequenced from the 5' end. After removing rRNA, as well as repetitive, mitochondrial and ambiguous sequences, 3,394 (81.4%) high-quality ESTs were obtained and further analyzed. Assembling of all available ESTs generated 961 cluster sets including 774 singleton (did not cluster with anything else) and 187 non-singleton clusters. They were deposited GenBank in NCBI with accession numbers: CD236662–CD240055. The average cluster contained 3.53 sequences. Of which, 757 (78.7%) and 123 (12.8%) were matched known genes and ESTs, respectively. The remaining 82 (8.5%) were considered as novel ESTs, because they did not match with any known genes or ESTs (Fig. 1A). The previous 5,877 ESTs that we generated from adult pituitary glands [15] were analyzed again and grouped into 2,747 clusters. Of which, 2,594 (87.0%), 344 (11.5%) and 45 (1.5%) were respectively identified as known genes, known ESTs and unique ESTs found only in the pituitary cDNA libraries (Fig. 1B). Although a total of 3,539 EST clusters were derived from human pituitaries, only 169 (4.8%) genes or ESTs were shared in both cDNA resources, which suggested that there might have been a significant switch of pituitary functions during development because of the differential gene expression profiles between fetal and adult pituitaries (Fig. 1C).

### Screening of developmentally relevant genes

Using the data from EST sequencing, we could distinguish the potential development-associated genes from the gene expression profiles, particularly, genes that were expressed highly in the human fetus. In the first 20 genes expressed highly in both fetal and adult pituitaries (Table 1), tissue-specific functional markers, such as GH, PRL, and glycoprotein hormones alpha (CGA), were frequently encountered in both tissues, which reflected the basic pituitary functions.

**Table 1:** The first 20 known genes expressed highly in human pituitaries based on EST data

**Unigene No**.	**Gene name**	**Copies**	%*
**Fetal pituitary**			
Hs.1905	prolactin+#	312	9.46
Hs.119689	glycoprotein hormones, alpha +#	146	4.43
Hs.115352	growth hormone 1+#	48	1.46
Hs.274460	olfactory receptor 5V1#	48	1.46
Hs.195453	ribosomal protein S27+#	24	0.73
Hs.75415	beta-2-microglobulin+#	22	0.67
Hs.70312	cytochrome c oxidase 7A2+#	15	0.46
Hs.315164	hypothetical protein#	14	0.42
Hs.83484	sex determining region Y-box 4#	8	0.24
Hs.7917	DKFZP564K247 protein#	7	0.21
Hs.273385	guanine nucleotide binding protein alpha+#	7	0.21
Hs.165590	ribosomal protein S13#	7	0.21
Hs.131814	tankyrase#	7	0.21
Hs.173912	eukaryotic translation initiation factor+	6	0.18
Hs.119222	suppression of tumorigenicity 13+#	6	0.18
Hs.177530	ATP synthase+	5	0.15
Hs.177656	calmodulin 1+	5	0.15
Hs.241429	cDNA DKFZp586C1923#	5	0.12
Hs.330994	RAB9#	4	0.12
Hs.46328	fucosyltransferase 2#	4	0.12
			
**Adult pituitary**			
Hs.115352	growth hormone 1+#	358	6.09
Hs.1905	prolactin+#	288	4.9
Hs.1897	proopiomelanocortin#	46	0.78
Hs.181165	eukaryotic translation elongation factor1#	45	0.77
Hs.273385	guanine nucleotide binding protein AS1+#	44	0.75
Hs.65149	growth hormone 2#	42	0.71
Hs.279789	glucose phosphate isomerase#	41	0.7
Hs.119689	glycoprotein hormones, alpha+#	37	0.63
Hs.77385	myosin, light polypeptide 6+#	30	0.51
Hs.278959	galanin-related peptide LOC51083#	25	0.43
Hs.247474	hypothetical protein FLJ21032#	21	0.36
Hs.180450	ribosomal protein S24#	20	0.34
Hs.155482	hydroxyacyl glutathione hydrolase#	19	0.32
Hs.76053	DEAD/H box polypeptide 5#	19	0.32
Hs.75360	carboxypeptidase E+#	18	0.31
Hs.112844	maternally expressed 3#	16	0.27
Hs.117950	phosphoribosylaminoimidazole carboxylase#	16	0.27
Hs.119598	ribosomal protein L3#	16	0.27
Hs.5464	skeletal muscle abundant protein#	16	0.27
Hs.179526	thioredoxin interacting protein #	15	0.26

* Percentage of a given EST in all ESTs obtained from that of cDNA libraries.

+ The genes presented in the EST libraries of both fetal and adult pituitary.

# Significant differences in gene expression (*P *< 0.05) between fetal and adult pituitaries .

Except for POU1F1, the pituitary-specific transcription factor, some development-associated genes, such as Sox4, suppression of tumorigenicity 13 (ST13) and fucosyltransferase 2, were highly expressed in fetal pituitary, which implies that these genes are involved in pituitary development. By comparing both cDNA resources, a total of 111 (3.1%) clusters were significantly differently expressed in fetal and adult pituitaries (*p *< 0.05) (See Additional file 1), including olfactory receptor 5, Hsp70-interacting protein, RAB9, heparin binding growth factor 8, EDNRA and mitogen-activated protein kinase phosphatase x, which were highly expressed in the fetus. Also, GA-bingding protein transcription factor (GABP), hypoxia-inducible factor 1 (HIF-1), and transcription factor DP3 (TFDP3) were presented in the fetal pituitary EST library.

### Expression pattern of Sox4

**Figure 2:**
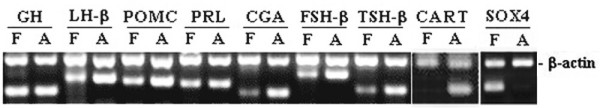
**Evaluating the expression pattern of selected known genes in fetal and adult pituitaries by semi-quantitative RT-PCR**. Relative transcriptional levels of some known genes, such as GH, PRL, POMC, CGA, TSH-β, LH-β, FSH-β, CART and SOX4 were analyzed by electrophoresis. F, fetal pituitary; A, adult pituitary.

**Figure 3:**
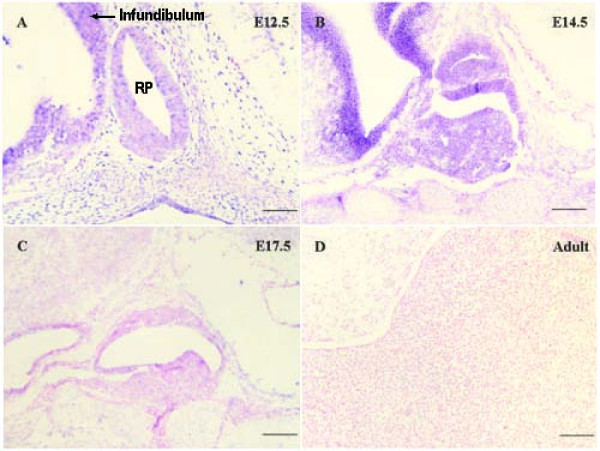
***In situ *hybridization of *Sox4 *in mice fetal pituitaries**. A-D show the images hybridized with Sox4 probe. The positive blue color was initiated by alkaline phosphatase and BCIP/NBT. All the slides were counterstained with 0.1% nuclear fast red after *in situ *hybridization. Original magnification, ×200 (bar, 50 μm).

There were eight (0.24%) transcripts for Sox4 in the fetal pituitary ESTs, but none was found in the adult pituitary cDNA library. The expression pattern of Sox4 during pituitary development was confirmed by semi-quantitative RT-PCR of human samples. The transcript level of Sox4 was much higher in fetal pituitary but was reduced to a very low level in adult pituitary (Fig. 2). At the same time, the transcription levels of GH, PRL, POMC, TSH, FSH, LH, CGA and CART (cocaine and amphetamine regulated transcript) were increased in adult samples. To further address the issue, we examined expression of Sox4 in E12.5, E14.5 and E17.5 mouse embryos and in adult pituitary using *in situ *hybridization. On E12.5, Sox4 was expressed in the pituitary at modest levels in the infundibulum and RP (Fig. 3A). By E14.5, Sox4 expression was most abundant in the undifferentiated cells of the anterior, intermediate and posterior lobes (Fig. 3B), but significantly decreased at E17.5 (Fig. 3C). In adults, the pituitary is fully differentiated and contains all of the hormone-secreting cell lineages. At this time-point, Sox4 was not detected in either the anterior, intermediate or posterior lobes (Fig. 3D). The expression pattern was similar to that of the gene in pancreas [16] and tibia [17] development, which indicates that the activity of this gene is restricted to the cell specification and terminal differentiation phase of pituitary development.

### Tissue expression patterns of novel ESTs

**Figure 4:**
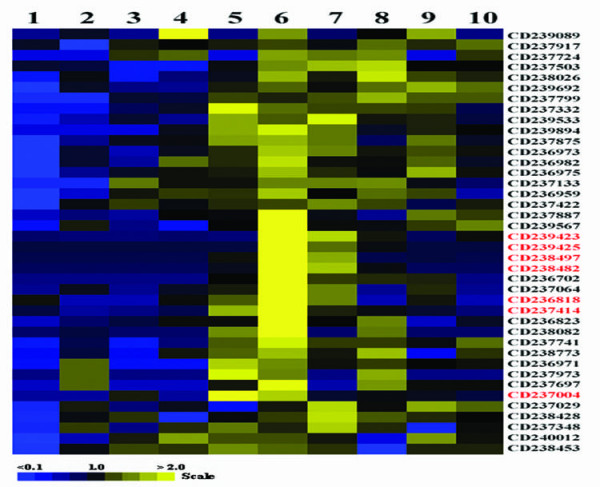
**Tissue expression patterns of 40 novel ESTs from human fetal pituitary**. Relative levels of novel ESTs in different tissues were evaluated by semi-quantitative RT-PCR, and were represented as color boxes with shades on a scale based on the average ratio of the density of PCR product band between the experimental gene and reference gene β-actin. The patterns were clustered and viewed using Gene Cluster and TreeView software (Stanford University). The relatively pituitary-specific ESTs were indicated by words in red. 1, heart; 2, spleen; 3, lung; 4, muscle; 5, brain; 6, fetal pituitary; 7, testis; 8, kidney; 9, thymus; 10, small intestine.

**Figure 5:**
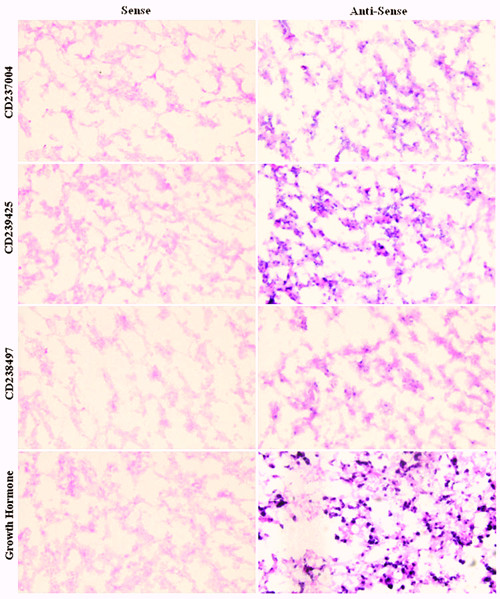
***In situ *hybridization of novel ESTs in human fetal pituitaries**. Expression of ESTs and GH as a control was detected by sense and anti-sense RNA probes, respectively. The positive blue color was initiated by alkaline phosphatase and BCIP/NBT. All the slides were counterstained with 0.1% nuclear fast red after *in situ *hybridization. Original magnification, ×400.

The present study generated 82 novel ESTs. They did not show homologous with any known genes/ESTs or proteins of any species but human genomic DNA by searching against nucleotide collection (nr/nt) database on NCBI. Also they are all presented as singleton clusters. To establish potential functions, the tissue expression patterns of 40 selected novel ESTs were evaluated by semi-quantitative RT-PCR (Fig. 4). Most of these ESTs were expressed in a limited manner in certain tissues, and not ubiquitously in all 10 tissues examined. Interestingly, some transcripts, such as CD239423, CD239425, CD238497 and CD238482, were highly expressed in pituitary glands and testes. Meanwhile, some ESTs, such as CD237004, CD236818 and CD237414 were specifically expressed in the brain, in addition to the pituitary. The expression of CD237004, CD238497 and CD239425 was further confirmed by *in situ *hybridization with human fetal pituitary tissues (Fig. 5). There were positive signals (blue spot) in all the sections hybridized with anti-sense probes.

## Discussion

Previous studies on the pituitary have focused on individual genes and signaling pathways involved in its development [4,18,19], as well as expression and release of pituitary hormones, such as PRL, LH and FSH [20,21]. With the advance of the human genome project, a large amount of genomic information is now available for surveying the physiological and pathological processes of humans at a global level. Hormonal genomics has been proposed to develop postgenomic approaches substituting gene-by-gene ones to reveal the subfamilies and pathways for genes involved in the hormones signaling [22]. The functional subgenomes of secreted extracellular signaling molecules, transmembrane receptors, intracellular signaling molecules, and transcriptional factors can now be analyzed in an integrated manner. Of which, EST sequencing, series analysis of gene expression (SAGE) and cDNA microarray or gene chip analysis are the most efficient strategies for profiling gene expression at the transcriptome level [23-25].

In this study, a total of 3,539 EST clusters were derived from 9,271 ESTs generated from cDNA libraries of human fetal and adult pituitaries. It was not as high as expected because of the limited number of human samples. We did confirm the information by semi-quantitative RT-PCR, *in situ *hybridization or reviewing previous studies. Aside from semi-quantitative RT-PCR in this study, microarray analysis and quantitative RT-PCR carried out in our previous study also showed that the expression level of hormones, such as TSHβ, POMC and GH in fetus, were relative lower than that in adult [11]. On the other hand, some genes such as EDNRA, were highly expressed in fetal pituitary, also in agreement with previous study performed by Dr. Ellestad using microarray [12]. Although these studies were all to highlight the gene expression profile of pituitary, there is only few genes overlapped between these screens. Most of the variations could be caused by methods different.

The partial transcription maps derived from these ESTs indicated that hormones and hormone-associated genes were predominant in adult pituitary, while development-associated genes were predominant in fetal pituitary, including Sox4 and ST13. Sox4 has been shown to be functionally involved in development of a wide range of tissues and cells, such as heart, B cells, reproductive system and central nervous system [26]. ST13 may facilitate the chaperone function of Hsc/Hsp70 in protein folding and repair, and in controlling the activity of regulatory proteins such as steroid receptors and regulators of proliferation or apoptosis [27,28]. POU1F1, necessary for cell lineage of thyrotrophs, somatotrophs and lactotrophs, was hit once in the cDNA library of fetal pituitary. No significant different of its expression level could be detected between fetal and adult pituitary. It is in agreement with that POU1F1 is necessary but not sufficient for hormones gene activation [29].

It was revealed for the first time that Sox4, a member of the SRY-like high-mobility group (HMG) box gene family, was one of the most abundant mRNAs in the fetal pituitary. The HMG box defines a superfamily of eukaryotic DNA-binding proteins of central importance in mammalian gene regulation [30], and SRY defines the mammalian testis-determining factor encoded by the Y chromosome [31]. Sox protein is a specific HMG-box factor that is similar to SRY. It is ubiquitous in the animal kingdom and is involved in diverse developmental processes, including germ layer formation, cell type specification, and organogenesis [32]. Sox4 null mice die during embryogenesis because of failure of endocardial ridge development, and blocking of B-cell lineage progression at the stage of pro-B-lymphocyte expansion [33].

Explanted fetal thymic organ cultures (FTOCs) of Sox-4-deficient thymus yielded 10–50-fold fewer CD4/CD8 double-positive and single-positive cells than FTOCs of littermates [34]. Lioubinski *et al *have reported that Sox4 is expressed in insulin-producing pancreas islets, and co-expressed with glucagon during tissue development [16]. Also, it has been reported that Sox4 is expressed in the mouse uterus under ovarian hormone control. This suggests a developmental role for this gene in the female reproductive system, because its expression always appears to be related to the maturational stage of the cell population [35]. Furthermore, Cheung *et al *[26] showed that Sox4 might indeed had another functional role in brain development. It has been demonstrated that Sox4 expression counteracts differentiation of radial glia and has to be down-regulated before full maturation can occur in the brain [36]. Radial glia with prolonged expression of Sox4 fail to migrate to the position normally assumed by Bergmann glia, and do not extend radial fibers toward the pial surface [37]. The expression pattern of Sox4 in pituitary development suggests that it is involved in pituitary cell differentiation; similar to its role in facilitating thymocyte differentiation, but it normally prevents premature differentiation of human pituitary.

In addition, we investigated the expression of 17 members of the Sox gene family in the fetal human pituitary using RT-PCR. Eleven of these members (Sox 2, 3, 4, 5, 7, 9, 10, 12, 13, 15 and 17) were detected (data not shown). It has recently been reported that Sox2 had a critical role in the development of the hypothalamo-pituitary axis [38,39], and deletion of Sox3 can lead to abnormal development of RP and defects in pituitary function [39,40]. Sox10 might be necessary for gonadotropin-releasing hormone cell differentiation [41]. Obviously, members of the Sox gene family might have a predominant role in the regulation of hypothalamus-pituitary axis formation.

Except for the genes known to be associated with pituitary development, our data suggest that some other genes are involved in the differentiation and maturation of human pituitary. They contained genes for cytokines and their receptors, transcription factors, and those involved in the cell cycle, DNA replication and signal transduction. Of which, GA-binding protein transcription factor is a critical and functionally relevant Ets factor that regulates rPRL promoter activity via the BTE site [42]. HIF-1 exerts an antiapoptotic role in HP75 in hypoxia [43]. TFDP3 was identified in pituitary for the first time, and only highly espressed in human testis, thymus and fetal pituitary according to our studies (data not show). It was reported as an inhibitor of E2F-induced apoptosis [44].

40 novel ESTs were produced in human fetal pituitary. They are all singletons which no EST was matched in a BLAST against the nonredundant EST database at NCBI. It is suggested that they are potential new genes, proteins and microRNAs that need to be investigated in the future. For example, CD239423 was located on Human DNA sequence from clone RP11-123K19 on chromosome 9, jointed by 13118–13292, 26649–26703, 29319–29386, and 39381–39729. The others such as CD239425, CD238497, CD237004 and CD238482 were found to reside in the introns of various genes. And their tissue expression patterns show that some novel ESTs exist particularly in fetal pituitary cells. It is indicated that EST sequencing is an efficient method for gene discovery and expression level analysis.

## Conclusion

In conclusion, the transcriptome approaches used in this study indicate some novel clues in human pituitary development. Of these, Sox4 and some novel ESTs are suggested to have important roles in pituitary development. Thus, the EST data was effective for identifying a comprehensive gene expression profile. It may lay the foundation for further investigation of the molecular mechanisms of human pituitary development and function.

## Methods

### Specimens

Two male fetal pituitaries were obtained from 16-week-old human fetuses, which were aborted accidentally without genetic, immunologic and infections diseases. The local ethical committee of Xijing hospital proved this study. The tissues were removed within 4 hours after abortion, frozen in liquid nitrogen, and stored at -80°C. C57BL/6 mouse embryos were obtained from the Animal Center of the Fourth Military Medical University. All human and animal subjects were treated according to relevant ethical criteria and guidance of the ethics committee in the university. Total RNAs of human adult heart, spleen, lung, muscle, brain, testis, kidney, thymus, small intestine and pituitary were bought from Clontech Company.

### Extraction of RNA

Total RNA was extracted using TRIZOL Reagent (Life Technologies), and treated with DNase I at 37°C for 30 minutes, then purified with phenol-chloroform. After extraction, the quality of total RNA was evaluated by electrophoresis on 1% agarose gel containing ethidium bromide, and the ratio of absorbance at 260/280 nm by spectrophotometer (Beckman).

### cDNA library construction and EST sequencing

A cDNA library from a 16-week-old male fetal pituitary gland was constructed by using the SMART cDNA Library Construction Kit (Clontech) according to the manufacturer's protocol. Briefly, 1.0 μg total RNA was used to synthesize the first strand with a modified oligo(dT) primer (CDS III/3' PCR Primer) and a short extended template (SMART IV Oligo) at 42°C for 1 hour. PCR amplification was carried out at 95°C for 5 seconds and 68°C for 6 minutes for 20 cycles using a PE9600 thermal cycler (Applied Biosystems). After treating 50 μl PCR products with 40 μg proteinase K at 45°C for 20 minutes to inactivate DNA polymerase, and purifying with phenol/chloroform/isoamylalcohol, the products were digested by *Sfi*I restriction enzyme at 50°C for 2 hours. The *Sfi*I-digested cDNA was ligated to the *Sfi*I-digested λTriplEx2 vector, and transformed into *Escherichia coli *BM25.8 after λ-phage packaging reaction. Bacterial colonies that contained cDNA inserts were randomly selected and grown, and plasmid extraction was performed in a 96-well format (Qiagen). DNA sequencing reactions were performed in a 9600 Thermal Reactor (Perkin-Elmer) using a Dye Primer Cycle Sequencing Kit (Perkin-Elmer). The DNA sequence reaction of each clone was taken from the 5' end with the PT primer (5'-ctc cga gat gtg gac gag c-3') on the 3700 Genetic Analyzer (PE Biosystems).

### EST database analysis and management

The qualified ESTs, which referred to those that contained < 3% ambiguous bases and were longer than 100 bp, were subjected to BLAST analysis. The EST was considered as part of a known gene or EST if it shared 95% homology with at least 100 bp of the known gene or EST, and the ESTs with no match to human ESTs were considered to be novel. All the ESTs were grouped into clusters of sequences which were thought to encode for one gene or EST, using the software CAT 3.5 (Pangea) with default parameters. The genes and ESTs were localized on chromosomes by searching the UniGene database  and genome information deposited in GenBank.

### Semi-quantitative RT-PCR

To confirm the expression and differentially transcriptional level of some selected genes/ESTs, a semi-quantitative RT-PCR was performed on fetal and adult RNA. The RT reactions were performed using SuperScript II RNase H reverse transcriptase (Invitrogen) according to the manufacturer's instructions. PCR amplification was carried out with specific primers that corresponded to the selected genes/ESTs, while β-actin was co-amplified as an internal control in the same reaction. A first cycle of 10 minutes at 95°C, 40 seconds at 55°C and 50 seconds at 72°C was followed by 40 seconds at 95°C for 30 cycles. The PCR products were analyzed on 2% agarose gels. The transcriptional levels of the genes were estimated according to the average ratio of density integration between the band density of specific genes and internal controls.

### *In situ *hybridization

5-μm tissue sections were made from paraffin blocks of C57BL/6 mouse embryos, and 10-μm sections were made from frozen human fetal pituitary tissue. Paraffin block tissue sections were dewaxed in xylene and dehydrated with increasing concentrations of ethanol, after mounting on glass slides. Digoxigenin (DIG)-labeled antisense RNA probes were prepared using DIG-UTP according to the standard protocol of the T7/SP6 RNA DIG Labeling Kit (Roche). All the procedures for *in situ *hybridization were performed as described previously [45], except for the following modification: hybridization was carried out at 60°C overnight, the concentration of the labeling probe was 1 ng/μl, and the slides were washed in 2 × SSC with 0.1 ng/ml RNase A at 37°C for 1 hour, followed by 2 × SSC at 60°C for 1 hour, and 0.2 × SSC at 60°C for 30 minutes. After blocking the slides, immunological detection for DIG with anti-DIG-AP was performed according to the instruction manual of the DIG Nucleic Acid Detection Kit (Roche). The color reaction was initiated by the addition of BCIP/NBT. Finally, the slides were counterstained with 0.1% nuclear fast red. The plasmid that contained Sox4 was kindly provided by Dr. Kaare M. Gautvik. All other probes were generated by RT-PCR. The following regions were used: mouse Sox4 (nt 30–408,), CD23847 (226–670), CD239425 (128–711), and CD237004 (88–653).

## Competing interests

The authors declare that they have no competing interests.

## Authors' contributions

YM conducted bioinformatics analysis, semi-quantitative PCR, *in situ *hybridization, and drafted the manuscript; XQ conducted the EST sequencing; JD conducted semi-quantitative PCR; SS, DF, JQ, XZ and XH participated in the DNA extraction, EST sequencing and RT-PCR; ZZ conducted bioinformatics analysis; XH and ZH served as primary investigator for the overall design and execution of the project.

## Supplementary Material

Additional file 1 **Supplementary Table 1 – Differentially expressed genes/ESTs between fetal and adult pituitary cDNA libraries.** Significant differences in gene expression between fetal and adult pituitaries were analyzed statistically. One hundred and eleven genes/ESTs were identified that had dominant roles in fetal or adult pituitaries. Click here for file
