# Supplementary material for: Identification of candidate genes for human pituitary development by EST analysis
Source: BMC Genomics. 2009 Mar 15;10:109. doi: 10.1186/1471-2164-10-109 (PMC2664823; doi:10.1186/1471-2164-10-109)
Supplement: Additional file 1 — Supplementary Table 1 – Differentially expressed genes/ESTs between fetal and adult pituitary cDNA libraries. Significant differences in gene expression between fetal and adult pituitaries were analyzed statistically. One hundred and eleven genes/ESTs were identified that had dominant roles in fetal or adult pituitaries. [file 1471-2164-10-109-S1.doc]

| **Supplementary Table 1. Differentially Expressed Genes/ESTs Between Fetal and Adult Pituitary cDNA Librares** (p<0.05)# | | | |
| --- | --- | --- | --- |
| **Unigene NO.** | **EST Copies**  **in fetal pituitary** | EST Copies **in adult pituitary** | **Gene Description** |
| **Genes/ESTs Expressed Highly in Fetal Pituitary** | | | |
| Hs.328613 | 171 | 0 | EST, Moderately similar to I60307 beta-galactosidase, alpha peptide |
| Hs.119689 | 146 | 37 | glycoprotein hormones, alpha polypeptide |
| Hs.301104 | 76 | 0 | ESTs, Moderately similar to 810024A ATPase 6 |
| Hs.316284 | 48 | 0 | EST, Weakly similar to 810024J URF 4 |
| Hs.274460 | 48 | 0 | olfactory receptor, family 5, subfamily V member 1 |
| Hs.327825 | 26 | 0 | EST, Weakly similar to 810024A ATPase 6 |
| Hs.195453 | 24 | 1 | ribosomal protein S27 |
| Hs.75415 | 22 | 10 | beta-2-microglobulin |
| Hs.300018 | 19 | 0 | EST, Moderately similar to 0512543A oxidase II,cytochrome |
| Hs.70312 | 15 | 1 | cytochrome c oxidase subunit VIIa polypeptide 2 |
| Hs.297184 | 15 | 0 | EST, Weakly similar to 810024J URF 4 |
| Hs.315164 | 14 | 0 | hypothetical protein similar to actin related protein 2/3 complex, subunit 5 |
| Hs.147587 | 12 | 0 | Homo sapiens mRNA; cDNA DKFZp547F134 |
| Hs.321770 | 10 | 0 | EST, Weakly similar to 810024C cytochrome oxidase I |
| Hs.331201 | 10 | 0 | EST, Weakly similar to 0512543A oxidase II,cytochrome |
| Hs.64753 | 10 | 0 | ESTs |
| Hs.164394 | 8 | 0 | ESTs |
| Hs.295945 | 8 | 0 | EST, Moderately similar to 810024C cytochrome oxidase I |
| Hs.296157 | 8 | 0 | EST, Weakly similar to 810024G URF 2 |
| Hs.83484 | 8 | 0 | SRY (sex determining region Y)-box 4 |
| Hs.7917 | 7 | 0 | DKFZP564K247 protein |
| Hs.165590 | 7 | 0 | ribosomal protein S13 |
| Hs.131814 | 7 | 0 | tankyrase, TRF1-interacting ankyrin-related ADP-ribose polymerase |
| Hs.119222 | 6 | 2 | suppression of tumorigenicity 13 (colon carcinoma) Hsp70-interacting protein |
| Hs.301809 | 6 | 0 | EST |
| Hs.330589 | 5 | 0 | EST, Weakly similar to 810024A ATPase 6 |
| Hs.332048 | 5 | 0 | ESTs, Moderately similar to 810024J URF 4 |
| Hs.330994 | 4 | 0 | RAB9, member RAS oncogene family |
| Hs.46328 | 4 | 0 | fucosyltransferase 2 (secretor status included) |
| Hs.241429 | 4 | 0 | Homo sapiens mRNA; cDNA DKFZp586C1923 |
| Hs.292378 | 4 | 0 | ESTs, Moderately similar to 810024E cytochrome oxidase III |
| Hs.299871 | 4 | 0 | ESTs, Weakly similar to I Chain I, Beta-Galactosidase (Chains I-P) |
| Hs.194382 | 4 | 0 | ataxia telangiectasia mutated |
| Hs.119192 | 4 | 0 | H2A histone family, member Z |
| Hs.101414 | 4 | 0 | KIAA0557 protein |
| Hs.44 | 4 | 0 | pleiotrophin (heparin binding growth factor 8, neurite growth-promoting factor 1) |
| Hs.324406 | 4 | 0 | ribosomal protein L41 |
| Hs.134829 | 3 | 0 | EST |
| Hs.229918 | 3 | 0 | EST, Moderately similar to 810024H URF 3 |
| Hs.237554 | 3 | 0 | cDNA FLJ14306 fis |
| Hs.273198 | 3 | 0 | EST, Moderately similar to 810024J URF 4 |
| Hs.306980 | 3 | 0 | clone IMAGE:3535910, mRNA |
| Hs.313829 | 3 | 0 | EST, Weakly similar to 2109260A B cell growth factor |
| Hs.320972 | 3 | 0 | ESTs, Weakly similar to 810024C cytochrome oxidase I |
| Hs.7446 | 3 | 0 | chromosome 6 open reading frame 5 |
| Hs.76252 | 3 | 0 | endothelin receptor type A |
| Hs.29106 | 3 | 0 | mitogen-activated protein kinase phosphatase x |
| Hs.16622 | 3 | 0 | zinc finger protein 185 (LIM domain) |
| **Genes/ESTs Expressed Highly in Adult Pituitary** | | | |
| Hs.115352 | 48 | 358 | growth hormone 1 |
| Hs.1897 | 0 | 46 | proopiomelanocortin (POMC) |
| Hs.181165 | 0 | 45 | eukaryotic translation elongation factor 1 alpha 1 (EEF1A1) |
| Hs.273385 | 7 | 44 | guanine nucleotide binding protein (G protein), alpha stimulating activity polypeptide 1 |
| Hs.65149 | 0 | 42 | growth hormone 2 (GH2) |
| Hs.279789 | 0 | 41 | glucose phosphate isomerase (GPI) |
| Hs.77385 | 4 | 30 | myosin, light polypeptide 6, alkali, smooth muscle and non-muscle |
| Hs.278959 | 0 | 25 | galanin-related peptide (LOC51083) |
| Hs.247474 | 0 | 21 | hypothetical protein FLJ21032 (FLJ21032) |
| Hs.180450 | 0 | 20 | ribosomal protein S24 (RPS24) |
| Hs.155482 | 0 | 19 | hydroxyacyl glutathione hydrolase (HAGH) |
| Hs.76053 | 0 | 19 | DEAD/H (Asp-Glu-Ala-Asp/His) box polypeptide 5 (RNA helicase, 68kD) (DDX5) |
| Hs.75360 | 3 | 18 | carboxypeptidase E |
| Hs.112844 | 0 | 16 | maternally expressed 3 (MEG3) |
| Hs.117950 | 0 | 16 | phosphoribosylaminoimidazole carboxylase, phosphoribosylaminoimidazole succinocarboxamide synthetase (PAICS) |
| Hs.119598 | 0 | 16 | ribosomal protein L3 (RPL3) |
| Hs.356428 | 0 | 16 | Homo sapiens mRNA expressed only in placental villi, clone SMAP83 |
| Hs.5464 | 0 | 16 | skeletal muscle abundant protein (SMAP) |
| Hs.179526 | 0 | 15 | thioredoxin interacting protein (TXNIP) |
| Hs.350108 | 0 | 15 | ribosomal protein, large, P0 (RPLP0) |
| Hs.232400 | 1 | 15 | heterogeneous nuclear ribonucleoprotein A2/B1 |
| Hs.118910 | 1 | 15 | tumor susceptibility gene 101 |
| Hs.182278 | 0 | 14 | calmodulin 2 (phosphorylase kinase, delta) (CALM2) |
| Hs.342389 | 0 | 14 | peptidylprolyl isomerase A (cyclophilin A) (PPIA) |
| Hs.74267 | 0 | 14 | ribosomal protein L15 (RPL15) |
| Hs.180859 | 0 | 13 | 16.7Kd protein (LOC51142) |
| Hs.279860 | 0 | 13 | tumor protein, translationally-controlled 1 (TPT1) |
| Hs.154704 | 0 | 12 | luteinizing hormone beta polypeptide (LHB) |
| Hs.169793 | 0 | 12 | ribosomal protein L32 (RPL32) |
| Hs.90336 | 1 | 12 | ATPase, H+ transporting, lysosomal (vacuolar proton pump), member J |
| Hs.350166 | 0 | 11 | ribosomal protein S6 (RPS6) |
| Hs.180877 | 0 | 10 | H3 histone, family 3B (H3.3B) (H3F3B) |
| Hs.2281 | 0 | 10 | chromogranin B (secretogranin 1) (CHGB) |
| Hs.275865 | 0 | 10 | ribosomal protein S18 (RPS18) |
| Hs.323468 | 0 | 10 | electron-transferring-flavoprotein dehydrogenase (ETFDH) |
| Hs.343411 | 0 | 10 | cDNA DKFZp586K2322 |
| Hs.10958 | 0 | 9 | RNA-binding protein regulatory subunit (DJ-1) |
| Hs.129952 | 0 | 9 | KIAA0560 gene product (KIAA0560) |
| Hs.14376 | 0 | 9 | actin, gamma 1 (ACTG1) |
| Hs.166975 | 0 | 9 | splicing factor, arginine/serine-rich 5 (SFRS5) |
| Hs.180946 | 0 | 9 | ribosomal protein L5 (RPL5) |
| Hs.279921 | 0 | 9 | hypothetical protein MGC8721 (MGC8721) |
| Hs.311296 | 0 | 9 | ESTs |
| Hs.350077 | 0 | 9 | ribosomal protein L21 (RPL21) |
| Hs.352556 | 0 | 9 | dimethylarginine dimethylaminohydrolase 1 |
| Hs.36927 | 0 | 9 | heat shock 105kD (HSP105B) |
| Hs.5722 | 0 | 9 | chromosome 1 open reading frame 28 (C1orf28) |
| Hs.62954 | 0 | 9 | ferritin, heavy polypeptide 1 (FTH1) |
| Hs.75258 | 0 | 9 | H2A histone family, member Y (H2AFY) |
| Hs.119122 | 0 | 8 | ribosomal protein L13a (RPL13A) |
| Hs.150580 | 0 | 8 | putative translation initiation factor (SUI1) |
| Hs.152519 | 0 | 8 | hypothetical protein FLJ20674 (FLJ20674) |
| Hs.16390 | 0 | 8 | hypothetical protein FLJ10035 (FLJ10035) |
| Hs.167791 | 0 | 8 | reticulocalbin 1, EF-hand calcium binding domain (RCN1) |
| Hs.349961 | 0 | 8 | ribosomal protein L6 (RPL6) |
| Hs.3776 | 0 | 8 | zinc finger protein 216 (ZNF216) |
| Hs.56205 | 0 | 8 | insulin induced gene 1 (INSIG1) |
| Hs.5662 | 0 | 8 | guanine nucleotide binding protein (G protein), beta polypeptide 2-like 1 (GNB2L1) |
| Hs.73965 | 0 | 8 | splicing factor, arginine/serine-rich 2 (SFRS2) |
| Hs.74137 | 0 | 8 | transmembrane trafficking protein (TMP21) |
| Hs.75914 | 0 | 8 | coated vesicle membrane protein (RNP24) |
| Hs.8997 | 0 | 8 | Sad1 unc-84 domain protein 1 (SUN1) |
| # Significant differences of gene expression *(P* < 0.05) between fetal and adult pituitaries by statistical analysis (http://igs-server.cnrs-mrs.fr). | | | |
